# Effect of isometric exercise on knee pain perception among old people with osteoarthritis

**DOI:** 10.6026/973206300221996

**Published:** 2026-04-30

**Authors:** Nikitaben Dahyabhai Patel, Arti Prasad Muley

**Affiliations:** 1 Department of Nursing, Parul University, Waghodia, Vadodara, Gujarat, India; 2 Department of Medicine, Parul Institute of Medical Sciences and Research, Faculty of Medicine, Parul University, Waghodia, Vadodara, Gujarat, India

**Keywords:** Osteoarthritis (OA), isometric exercise, pain perception, old people

## Abstract

Knee osteoarthritis (OA) has been identified as among the top three causes of chronic pain and functional limitations in aging populations. The first line of treatment for OA is exercise as part of a conservative management plan. Therefore, it is of interest to determine if isometric exercise would reduce pain perception of the knee in elderly patients (age 60-75) with knee OA. The sample consisted of 40 participants matched to experimental or control groups using consecutive sampling. Pain was measured before and after the intervention using a standardized pain scale. After the intervention, the mean pain score for the experimental group decreased from 4.70 to 0.37, for an overall mean difference of 4.32 (p < 0.05). Thus, we report that isometric exercise of the knee for the treatment of knee OA significantly reduces pain perception in older adults with knee OA.

## Background:

Osteoarthritis is a long-term degenerative joint problem that has caused many older adults to become disabled [1]. The knee is the most typical location for developing osteoarthritis [2]. Osteoarthritis is more common as people age and reduces mobility as well as quality of life [3]. Osteoarthritis damages a joint in three ways by degenerating cartilage, by creating synovial fluid inflammation and by decreasing a person's ability to regenerate the joint [4]. These physical changes to joints create pain, stiffness and limitation of physical function. Osteoarthritis risk factors include being older, being obese, having had an injury to the joint and having weak muscles [5]. A sedentary lifestyle will also increase the rate at which osteoarthritis is developing in an individual. Current treatment guidelines for osteoarthritis recommend that non-pharmacological methods be used first to treat osteoarthritis [6]. Exercise is recommended for decreasing joint pain and increasing functional capacity [7]. Strengthening exercises can also be helpful in improving joint stability and decreasing stress on the joint. Isometric exercises are a type of exercise where the muscles are contracted without any movement of the joint and they do not put any stress on the joint [8]. Isometric exercises are safe and appropriate for elderly individuals who have limited physical mobility and who do not need to use any special equipment [9]. Isometric exercises can be performed easily in the home or the community [10]. Therefore, it is of interest to investigate the effect of isometric exercise on the perception of knee pain in older adults who have osteoarthritis.

## Materials and Methods:

The researcher used a quantitative method to investigate how effective isometric exercises are in reducing the perception of knee pain in older adult patients diagnosed with osteoarthritis. The study utilized a pre experimental research design-one group pre-test & post-test design and was completed within specified areas in Daman. In total, 40 elderly individuals participated in this study, which utilized a non-probability consecutive sampling technique to identify eligible subjects. Participants had to be between 60 and 75 years of age; both genders were included, with the additional criterion being if they experienced knee pain due to osteoarthritis and they were willing to take part in the study. Elderly persons with extreme restrictions in movement, persons who had undergone knee surgery in recent months and/or those suffering from other inflammatory joint problems were not eligible for inclusion in this study. Following approval from the Institutional Human Ethics Committee, all of the participants signed a written informed consent prior to the data collection process. After baseline demographic information and a standardized pain scale were recorded, the experimental group was given a defined form of isometric exercise treatment for their knee joints according to established standards; meanwhile, the control group continued receiving standard care. Following the completion of the exercise intervention period, post-test assessments of the participants' knee pain were conducted. All data collected were analyzed using descriptive and inferential statistical techniques and descriptive statistics were generated to summarize the data collected in forms of frequency, percentage, mean and standard deviation, along with testing of the hypotheses using Inferential Statistics.

## Results:

The findings of this research study provide an overview of several different demographic variables relating to each of the participants as well as the levels of pain that each participant experienced both before and after an exercise program consisting of only isometric type exercises. This includes a comparison of average pain levels prior to and following participation in the exercise program as well as evidence of the effectiveness of an isometric exercise program to reduce pain in older adults who have osteoarthritis. In addition, the demographic data collected from the 40 elderly participants includes the pre-test pain perception levels and post-test pain perception level data, including the mean difference between these two measurements. Also, within this listing the effectiveness of the isometric exercise program via the use of inferential statistics (t-tests). Other representation formats may be used to represent these findings as long as they demonstrate a wide range of possible distributions and associations by comparing the sub-group categories of pain level versus all other variables, as well as how each of the variables is related to each other. All results will be displayed in a logical manner and with alternating grey and white rows to allow for increased visibility and understanding of the findings. The study on the elderly included 40 people, as shown in Table 1 (see PDF), which breaks down the participants' ages into different categories. Most of the elderly, 33 (82.5%), were aged 60-65 and there were 6 (15.0%) aged 66-70, with just 1 participant aged 71-75 (2.5%). This indicates that the majority of participants fell within the younger elderly range. Table 2 (see PDF) outlines how the gender of the participants was distributed amongst males and females. Of the 40 elderly participants, the significant majority of the sample, 32 (80.0%), were male with 8 (20.0%) female. This indicates a significant imbalance between the numbers of males and females in the sample, which may point to occupational risk factors and lifestyle choices being responsible for knee osteoarthritis in this sample population. Table 3 (see PDF) details how participants were divided according to other demographic characteristics. All 40 (100%) participants had received education through either primary or secondary schools. Of the different types of previous occupations, the largest group, 26 (65.0%), were labourer and 7 (17.5%) were homemakers, followed by 5 (12.5%) company workers and 2 (5.0%) watchmen. Based on the calculation of body mass index, 23 (57.5%) participants fell between 25-29.9 (overweight) and 17 (42.5%) fall within the normal range for BMI. No participant reported a family history of osteoarthritis nor had participants participated in any kind of sports prior to the study. A mixed diet was consumed by 25 (62.5%) participants and 26 (65.0%) participants were sedentary in their lifestyles. Importantly, none of the participants had any type of regular exercising program. Knee pain levels were compared before and after an isometric exercise intervention in Table 4 (see PDF). The majority of participants in the pre-test, 39 (97.5%), reported moderate levels of pain, while 1 (2.5%) reported mild pain and none reported no pain at all. Conversely, after completing the isometric exercise program, 25 (62.5%) participants no longer reported any pain and 15 (37.5%) experienced mild pain. Furthermore, none of the participants reported moderate levels of pain after completing the intervention. Therefore, the isometric exercise intervention greatly improved participants' perceptions of their knee pain. Using Table 5 (see PDF), the average pain scores before and after the intervention can be compared. Before the exercise program, the average pain score was 4.70 with a standard deviation of 0.82 (moderate levels of pain), while the average pain score after the intervention dropped significantly to 0.37 with a standard deviation of 0.49. The average difference in pain scores between pre and post was 4.32, indicating that the intervention reduced the participant's knee pain very significantly. Table 6 (see PDF) compares the effectiveness of isometric exercise against knee pain perception based upon inferential statistics. The t statistic was calculated as 29.83 with 39 degrees of freedom, which is statistically significant at p < .05. This indicates that the pre-test average pain score was significantly higher than the post-test average pain score. Therefore, the research supports the conclusion that using isometric exercises reduce knee pain significantly.

## Discussion:

Knee osteoarthritis (OA) is a primary cause of chronic pain and disability in older adults. The main objective of all conservative treatment options for knee OA will be to reduce pain and enhance function. Exercise has been established to be one of the first modes of treatment for knee OA and is supported as a first-choice approach [11]. This study supports the claim of a statistically significant reduction in knee pain status after a period of isometric exercise. At the baseline, most participants reported moderate levels of pain. Upon completion of the exercise program, the majority of participants reported no pain or only mild levels of pain. These results support the use of strengthening exercises for pain management [12]. Performing isometric exercises will strengthen the muscles and provide stability. By having increased strength in the quadriceps, this can decrease the amount of mechanical load placed on the knee joint. Consequently, decreased load on the knee means less stress placed on the joint and thus a lower perception of pain and increased ability to perform functional activities [13]. Furthermore, the mean amount of pain reduction from the exercise program is similar to that found in previous research of other exercise programs in this clinical population. There is ample evidence in the extant literature that structured exercise programs have produced similar benefits for older adults with knee OA compared to the results of this study [14]. Exercise therapy also demonstrates an increase in confidence and increased levels of activity. The demographic data revealed a high prevalence of sedentary lifestyles and lack of regular physical activity. This is critical in emphasizing the need for simple and accessible options. Isometric exercises can be performed inexpensively in the home, community-based programs or group settings [15]. The findings from this study support the current clinical guidelines recommending exercise therapy as a fundamental strategy for treating OA. Therefore, including isometric exercise into routine treatment for patients may reduce the need for pharmacological treatment for knee OA [16]. In addition, the results of this study provide evidence supporting the positive effect of isometric exercise within the elderly population in the community. This research demonstrates that isometric strengthening exercises can provide significant reduction in knee pain perception. The limitations of this study were the small sample size and lack of randomization, yet these limitations still support the value of low-cost forms of exercise interventions within a community setting.

## Conclusion:

In older individuals experiencing osteoarthritis, isometric exercise has a positive effect on reducing their pain from the knees. There can be significant increases in the quality of life and mobility in older adults, as a result of the encouraged routine of structured strengthening exercises.
